# Metabolic profiling and gene expression analyses provide insights into cold adaptation of an Antarctic moss *Pohlia nutans*

**DOI:** 10.3389/fpls.2022.1006991

**Published:** 2022-09-13

**Authors:** Shenghao Liu, Tingting Li, Shuo Fang, Pengying Zhang, Dan Yi, Bailin Cong, Zhaohui Zhang, Linlin Zhao

**Affiliations:** ^1^Key Laboratory of Marine Eco-Environmental Science and Technology, First Institute of Oceanography, Ministry of Natural Resources, Qingdao, China; ^2^School of Advanced Manufacturing, Fuzhou University, Jinjiang, China; ^3^National Glycoengineering Research Center, School of Life Sciences, Shandong University, Qingdao, China; ^4^Laboratory for Marine Ecology and Environmental Science, Pilot National Laboratory for Marine Science and Technology, Qingdao, China

**Keywords:** cold stress, flavonoids, very-long-chain fatty acids, metabolomic profiling, transcriptomic sequencing, bryophytes

## Abstract

Antarctica is the coldest, driest, and most windy continent on earth. The major terrestrial vegetation consists of cryptogams (mosses and lichens) and two vascular plant species. However, the molecular mechanism of cold tolerance and relevant regulatory networks were largely unknown in these Antarctic plants. Here, we investigated the global alterations in metabolites and regulatory pathways of an Antarctic moss (*Pohlia nutans*) under cold stress using an integrated multi-omics approach. We found that proline content and several antioxidant enzyme activities were significantly increased in *P. nutans* under cold stress, but the contents of chlorophyll and total flavonoids were markedly decreased. A total of 559 metabolites were detected using ultra high-performance liquid chromatography/electrospray ionization tandem mass spectrometry (HPLC-ESI-MS/MS). We observed 39 and 71 differentially changed metabolites (DCMs) after 24 h and 60 h cold stress, indicating that several major pathways were differentially activated for producing fatty acids, alkaloids, flavonoids, terpenoids, and phenolic acids. In addition, the quantitative transcriptome sequencing was conducted to uncover the global transcriptional profiles of *P. nutans* under cold stress. The representative differentially expressed genes (DEGs) were identified and summarized to the function including Ca^2+^ signaling, ABA signaling, jasmonate signaling, fatty acids biosynthesis, flavonoid biosynthesis, and other biological processes. The integrated dataset analyses of metabolome and transcriptome revealed that jasmonate signaling, auxin signaling, very-long-chain fatty acids and flavonoid biosynthesis pathways might contribute to *P. nutans* acclimating to cold stress. Overall, these observations provide insight into Antarctic moss adaptations to polar habitats and the impact of global climate change on Antarctic plants.

## Introduction

The Antarctic continent and surrounding islands occupy a terrestrial area of 13.95 million square kilometers and offer some of the world’s coldest and most arid environments (Block et al., 2009; Convey et al., 2014). The extremely cold temperature is likely one of most striking features in Antarctica, causing extensive snow and ice cover, short growing seasons, and often little flora cover (Convey et al., 2018). In contrast, the climates of sub- and maritime- Antarctic regions are relatively milder (Perera-Castro et al., 2020). For example, at four observing sites of the Antarctic peninsula, annual mean air temperatures range from −10.6 to −3.9°C with minimum and maximum values from −47.5°C to + 11.2, while the annual mean ground temperatures range from −9.0 to −1.7°C with minimum and maximum values from −39.2°C to + 24.3, respectively (Convey et al., 2018). Summer daytime temperatures on the South Shetlands Islands range from −5°C to 13°C, and winter daytime temperatures can drop as low as −30°C (Perera-Castro et al., 2021). There are 73 days per year when the average temperature is above 0°C on the Antarctic peninsula, along with a higher frequency of air freeze-thaw cycles during the austral summer (Convey et al., 2018). These annual and shorter-term temperature patterns for terrestrial biota are representative of the range of habitats within the full latitude of the Antarctic peninsula (Convey et al., 2018). Consequently, Antarctic terrestrial plants are forced to live in cold environments for the majority of their lives, with quick narrow windows of near-optimal conditions (Perera-Castro et al., 2020).

Mosses, lichens and only two vascular plant species dominate the Antarctic terrestrial vegetation (Skotnicki et al., 2000; Amesbury et al., 2017; Cannone et al., 2022). They are nearly restricted to seasonally or permanently snow- and ice-free areas accounting for ∼3.4% of the Antarctic continent, where sufficient summer snowmelt occurs (Convey et al., 2014; Lee et al., 2017; Convey and Peck, 2019). From Alexander Island (69.4°S) to Elephant Island (61.1°S) and northeast to South Orkney Islands (60.7°S), moss banks can be found sparsely throughout the western Antarctic Peninsula (Amesbury et al., 2017). Due to the rapid warming of Antarctic Peninsula, moss growth or accumulation rates have shown a significant increase over the last 50 years (Amesbury et al., 2017; Convey and Peck, 2019). In contrast, the Windmill Islands in East Antarctica are habitat to some of the best-developed and massive moss ecosystems, whereas the health of moss-beds is declining due to the recent climate changes of colder summer and drying climate (Clarke et al., 2009; Wasley et al., 2012; Malenovský et al., 2017; Robinson et al., 2018). These native terrestrial plants normally grow at the survival limitations and are particularly susceptible to environmental changes (Convey and Peck, 2019; Perera-Castro et al., 2021). They can serve as sensitive environmental indicators that can be used to detect subtle changes in Arctic and Antarctic conditions (Malenovský et al., 2017). On the other hand, since these native plants have adapted to the region’s harsh environments over many millions of years (Convey and Peck, 2019), they therefore inevitably evolve a variety of strategies, ranging from molecular to whole cell, as well as ecosystem levels, to sustainably inhabit these low-temperature conditions. For example, Antarctic moss banks have surface temperatures considerably above air temperatures of over 15°C, relying on the water content of moss tundra (Perera-Castro et al., 2020). The ability of Antarctic moss to enhance photosynthesis for a short summer and minimize respiratory carbon losses is crucial for survival in this frigid environment with frequent freezing and thawing cycles (Lovelock et al., 1995; Perera-Castro et al., 2020; Perera-Castro et al., 2021). The Antarctic mosses are characterized by considerable physiological and ecological flexibility, and it is expected to show increases in species diversity, population sizes, and geographic ranges (Convey and Peck, 2019). Notably, mosses have well-developed stress tolerance traits. Their gametophytes can survive in permafrost for long periods of time and regrowth directly from millennial-scale preserved material (Roads et al., 2014). However, their genomic and metabolic features as well as adaptation processes to this extreme cold environment remain poorly understood.

Plants are increasingly subjected to the dramatic temperature fluctuations due to global climate changes and the accompanying extreme weather incidents (Ding and Yang, 2022). Cold stress restricts the geographic distribution of plants and dramatically affect plant growth, development, and crop yields (Shi et al., 2018; Zhang et al., 2020). Plants have evolved various strategies for sensing and responding to cold stress. How plants sense cold signals, however, is still a major fundamental subject that has to be addressed. Plants may perceive and transmit cold signals to cells *via* a variety of mechanisms, including calcium (Ca^2+^), reactive oxygen species (ROS) and plant hormone signaling (Ding et al., 2020; Adhikari et al., 2022). Cold-responsive genes will be activated and regulated at transcriptional and posttranscriptional levels in order for plants to withstand cold stress (Ding and Yang, 2022). For example, C-repeat/DREB binding factors (CBFs) and heat shock factors (HSFs) have been identified as important transcription factors that regulate the expression of cold-responsive genes (Shi et al., 2018), while alternative splicing, a posttranscriptional regulatory process in which an intron-containing gene generates more than one mRNA variant, is also important for cold-stress tolerance (Ling et al., 2021; Ding and Yang, 2022). Cold-stress responses are triggered to beneficially aid plant survival, but they generally generate growth inhibition by repressing cell division and expansion (Zhang et al., 2020; Ding and Yang, 2022). Cold-induced inhibition of growth is a subsequent strategy of limits in energy/carbon supply and active suppression of growth signaling pathways, which allows plants to efficiently defend themselves against adverse environments (Zhang et al., 2020). Cold acclimation is a phenomenon observed not only in angiosperms but also in other classes of land plants. Bryophytes represent the basal land plant lineage. The bryophyte gametophytes that grow in natural habitats possess greater freezing tolerance, even though the level of tolerance varies among species (Rütten and Santarius, 1992). The moss *Physcomitrella patens* stimulates an abscisic acid-dependent signaling process in freezing tolerance, involving the action of ABI3 transcription factor (Bhyan et al., 2012; Tan et al., 2017). In addition, the global transcriptomic analysis of the moss *P. patens* under cold stress reveal that the early response is dominated by orphan genes encoding yet unknown acclimation processes (Beike et al., 2015). Currently, the metabolic and transcriptional features underlying the adaptation of bryophytes to cold stresses are still less documented.

We infer that the ability of Antarctic mosses adapting to the extreme habitats is likely relied on a set of distinct metabolites and different functional genes corresponding to stress environments. Here, we conducted an integrated metabolomics and transcriptomics approach to reveal global properties of the Antarctic moss *Pohlia nutans* under cold stress. A total of 559 metabolites were detected with 39 and 71 significantly changed metabolites (SCMs) in two comparison groups (Cold_24 h vs. CK and Cold_60 h vs. CK), respectively. Furthermore, the representative differentially expressed genes (DEGs) were identified and classified into different classes including Ca^2+^ signaling, plant hormone signaling pathways, fatty acids biosynthesis, flavonoid biosynthesis, antioxidant enzymes, transcription factors, and other gene families, as well as several novel gene families related to cold stress. We also investigated the interaction of cold signaling (jasmonate, Auxin, and CBFs) and metabolite biosynthesis (fatty acid and flavonoids) pathways as well as potential regulatory genes involved in cold responses. Overall, our results shed light on the metabolic pathways and candidate genes underlying the adaptations of this basal land plant to the polar terrestrial environments.

## Experimental procedures

### Plant samples and cold stress treatments

The moss samples were collected from the vicinity of the Great Wall Station in the Fildes Peninsula of Antarctica. The moss *Pohlia nutans* isolate NO.L was separated and purified from the mixed field samples. In our laboratory, *P. nutans* was then cultivated on a substrate mixture of Pindstrup substrate (Pindstrup Mosebrug A/S, Ryomgaard, Denmark) and regular soil (ratio 1:1) at 16°C, 70 μmol photons⋅m^–2^⋅s^–1^ continuous light in an incubator (GXZ-500, Ningbo Jiangnan Instrument, Ningbo, China). Plants were always covered by transparent plastic film to prevent moisture loss. For the cold treatments, two-month-old plants were transferred to 0°C and incubated for 6 h, 24 h and 60 h. The gametophytes were cut and frozen with liquid nitrogen. These samples were used for transcriptome sequencing and LC-MS/MS analysis. The plants without cold stress treatments were collected and used as control group. Three biological replicates were collected from different flowerpots.

### Measurement of proline and malondialdehyde contents and antioxidant enzyme activities

Mosses were placed under 0°C in a temperature controlled light incubator for 0 h, 12 h, 24 h, and 60 h. Then, the moss gametophytes with different treatment times were cut and ground into powders by freezing them in liquid nitrogen. The biochemical features of the plants were detected using commercial kits (Nanjing Jiancheng Bioengineering Institute, Nanjing, China). Briefly, 0.1 g of ground sample powder was used and a 10% tissue homogenate was obtained by adding the powder to the appropriate extracting buffer. The supernatant was collected and used for each biochemical determining according to the kit instructions. Proline quantitation was achievable by reaction with ninhydrin (Product No. A107-1-1). Malondialdehyde (MDA) content was measured by the thiobarbituric acid assay (Product No. A003-2-3). Peroxide (POD) catalyzed the reaction of hydrogen peroxide (H_2_O_2_) and the enzyme activity was calculated by measured the absorbance changes at 420 nm (Product No. A084-3-1). Superoxide dismutase (SOD) activity was determined by a xanthine-xanthine oxidase-nitro blue tetrazolium assay (Product No. A001-3). Catalase (CAT) activity was detected using the colorimetric ammonium molybdate method (Product No. A007-1-1). Ascorbate peroxidase (APX) is mainly found in chloroplasts, and its activity is measured by catalyzing ascorbic acid’s reaction with H_2_O_2_ (Product No. A123-1-1). All of the above experiments were repeated three times.

### Widely targeted metabolome analysis

The moss gametophytes from cold stress groups (i.e., Cold_24 h and Cold_60 h) and control groups (i.e., CK) were cut and used for metabolite analysis. The metabolite extraction, qualitative and quantitative analysis were performed in accordance with previously described methods (Li et al., 2021; Liu et al., 2021; Wang et al., 2021b). Briefly, the freeze-dried samples were ground to powder using a mixer mill (MM 400, Retsch, Laichi, Germany). Then, 0.2 g of the powder was extracted with 70% aqueous methanol (1.2 mL) at 4°C overnight. The extraction mixtures were vortexed six times during incubation for efficient extraction. The extracts were centrifuged at 10,000 *g* for 10 min, and the supernatant was filtered and used for metabolite analysis. The ultra-performance liquid chromatography (UPLC) (Shim-pack UFLC SHIMADZU CBM30A, Kyoto, Japan) and tandem mass spectrometry (MS/MS) (SCIEX QTRAP 6500, Applied Biosystems, Framingham, MA, United States) were used for metabolite identification. The metabolites were determined by primary and secondary spectral properties according to the public metabolite database and the self-built database MWDB V2.0 (5,000 + compounds, Metware Biotechnology Co., Ltd., Wuhan, China). Metabolites were quantified *via* the multiple reaction monitoring mode (MRM) using triple quadrupole mass spectrometry. Quality control (i.e., mix) prepared by mixing extract samples were parallelly conducted to monitor the reproducibility of detection process.

### RNA isolation and unique molecular identifier library preparation

Total RNA was extracted with TRIzol reagent (Invitrogen, Carlsbad, CA, United States). RNA degradation was detected using 1% agarose gel electrophoresis, while RNA purity and concentration were determined using NanoPhotometer^®^ spectrophotometer (Implen, München, Germany). mRNA was then captured and purified from total RNA with mRNA Capture Beads (Vazyme Biotech, Nanjing, China). After fragmenting the mRNA into 100∼200 nt in the Fragmentation Buffer, mRNA was reverse-transcribed into cDNA. Synthesized cDNA was purified using DNA Clean Beads (Vazyme Biotech).

We used a sequencing strategy with optimized single-molecule barcodes to minimize sequence dependent bias and amplification noise (Shiroguchi et al., 2012; Ogawa et al., 2017). The Purified cDNA was ligated to the adaptor with UMI (Unique molecular identifier) and purified using DNA Clean Beads. Finally, cDNA libraries were constructed from cDNA using PCR amplification (NEBNext Ultra RNA Library Prep Kit, Ipswich, MA, United States). The qualities of cDNA libraries were detected by the Agilent Bioanalyzer 2100 (DNA Technologies Core, Davis, CA, United States).

### Transcriptome sequencing, sequence assembly, and functional annotation of differentially expressed genes

The libraries were subjected for sequencing on Illumina novaseq 6000 Platform (each with 6G raw data), and generated 150 nt pair-end reads. The raw reads of FASTQ format were first processed using in-house scripts to remove reads containing adapters, reads containing ploy-N and low-quality reads. Clean reads with high-quality were then retrieved and used for downstream analyses. UMI reads were identified using UMI-tools v1.0.0 (Smith et al., 2017). To identify the duplicate reads, UMIs were first removed from each read, and the remaining parts were mapped to the reference genome using Hisat2 v2.0.4 (Kim et al., 2015; Kim et al., 2019). Reads mapping to the same location on the reference genome were identified as duplicates. Following that, duplicate reads with the same UMI were identified as non-natural duplications, which were then removed from the clean data.

The paired end clean reads were aligned to the reference genome using Hisat2. The reads for each gene were counted with HTSeq v0.6.1 (Anders et al., 2015). The gene expression levels were estimated based on expected number of fragments per kilobase of transcript sequence per million base pairs sequenced (FPKM) (Zhao et al., 2020; Zhao et al., 2021). The differential expression analysis was analyzed using the DESeq R package v1.10.1 (Wang et al., 2010), and the differential expression genes (DEGs) were identified with a threshold of adjusted *P*-value < 0.05. Gene functions were annotated based on BLAST alignment against the nr and Swiss-Prot databases. Gene Ontology (GO) enrichment analysis was performed using GOseqR package, while KEGG pathway enrichment analysis was implemented with KOBAS software (Mao et al., 2005).

### Quantitative real-time RT-PCR analysis

We conducted a quantitative real-time RT-PCR analysis (qPCR) analysis to validate the expression levels of differentially expressed genes in transcriptome sequencing. The moss gametophytes were used to isolate total RNA, and 0.5 μg of that RNA was used to synthesize the first-strand cDNA with *TransScript*^®^ All-in-One First-Strand cDNA Synthesis SuperMix for qPCR with One-Step gDNA Removal Kits (Transgen Biotech Company, Beijing, China). We assessed the expression stability of GAPDH (Poh0204970.1), Actin 1 (Poh0314480.1), and tubulin beta-1 chain (Poh0012540.1) genes under cold stress. The GAPDH gene was identified as the best reference gene to normalize the template. The gene specific primers were listed in Supplementary Table 1. qPCR analysis was performed using *PerfectStart*^®^ Green qPCR SuperMix Kits (Transgen) on a LightCycler96 qPCR instrument (Roche, Switzerland). The cycling regime is 94°C for 30 s, followed by 40 cycles of amplification (94°C for 10 s, 58°C for 15 s, and 72°C 10 s). The relative gene expression levels were calculated using the comparative Ct (2^–Δ^ ^Δ^ *^Ct^*) method (Livak and Schmittgen, 2001). The experiments were carried out using three biological replicates from three different experiments.

### Statistical data analysis

All data were presented as the mean ± SD. All experiments were performed in three biological replicates. For quantitative analysis, results were statistically compared between different groups and the statistical significance were calculated using Student’s *t* test (*P < 0.05, ^**^P < 0.01). For multivariate data analysis of metabolome analysis, the raw data signals were processed using the Analyst 1.6.3 software (AB Sciex, Framingham, MA, United States). The original abundance of metabolites was log-transformed to normalize the data and for the homogeneity of between-study variance. Principal component analysis (PCA), hierarchical clustering analysis (HCA), and orthogonal projections to latent structure-discriminant analysis (OPLS-DA) were performed using R^1^ for the analysis of metabolite cultivars-specific accumulation (Nyamundanda et al., 2010; Li et al., 2021). A permutation test (200 permutations) was conducted to avoid overfitting. Variable importance in projection (VIP) values of all metabolites from the OPLS-DA model were calculated to determine the relative importance of metabolites. Metabolites with fold change ≥ 2 or fold change ≤ 0.5 and VIP ≥ 1 were considered as differential metabolites between two groups (Cold_24 h vs. CK, Cold_60 h vs. CK).

## Results

### Morphological and biochemical changes under cold stress

**FIGURE 1:**
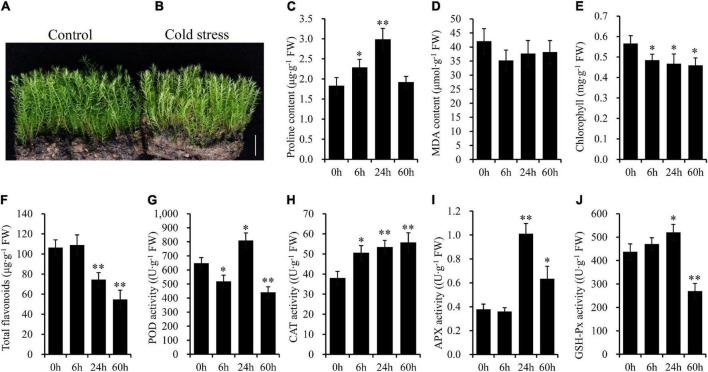
The moss *Pohlia nutans* has a potential capability of tolerance to cold stress. **(A)** Photo of moss gametophytes grown under normal condition. **(B)** Photo of moss gametophytes placed under 0°C for 60 h to carry out cold stress. **(C–J)** The biochemical features of mosses under cold stress. All experiments were repeated three times. Significant difference (*P < 0.05, ^**^P < 0.01). Scale bar = 1.0 cm.

The moss gametophytes of *P. nutans* were green when cultured under standard conditions (Figure 1A). When mosses were placed under cold stress, the color of the gametophytes became light green (Figure 1B). However, there was no other phenotypic difference between the cold-treated groups and the control groups under cold stress. To detect the response of mosses to cold stress, we conducted biochemical experiments on control samples and cold-treated samples at different time points. Proline content was significantly increased at 6 h and 24 h after cold stress (Figure 1C). The MDA content, indicating lipid peroxidation in cell membrane, was slightly decreased under cold stress (Figure 1D). The chlorophyll content was significantly decreased under cold stress (Figure 1E). Similarly, the contents of total flavonoids were also markedly decreased 0.70-fold after 24 h, and 0.52-fold after 60 h of cold stress (Figure 1F). When *P. nutans* was subjected to cold stress, the POD activity decreased by 0.80-fold at 6 h, increased by 1.25-fold at 24 h, and decreased by 0.68-fold at 60 h (Figure 1G). The CAT activity was markedly increased in *P. nutans* under cold stress (Figure 1H). The APX activity was significantly increased by 2.68-fold at 24 h and 1.68-fold at 60 h of cold stress (Figure 1I). The SOD activity decreased at 6 h after cold stress treatment, but significantly increased by 1.35-fold and 1.33-fold at 24 h and 48 h, respectively (Figure 1J). Therefore, *P. nutans* actively respond to cold stress with the increased content of proline and enhanced antioxidant enzyme activities.

### Metabolite profiling and differentially changed metabolites identification of *Pohlia nutans* under cold stress

**FIGURE 2:**
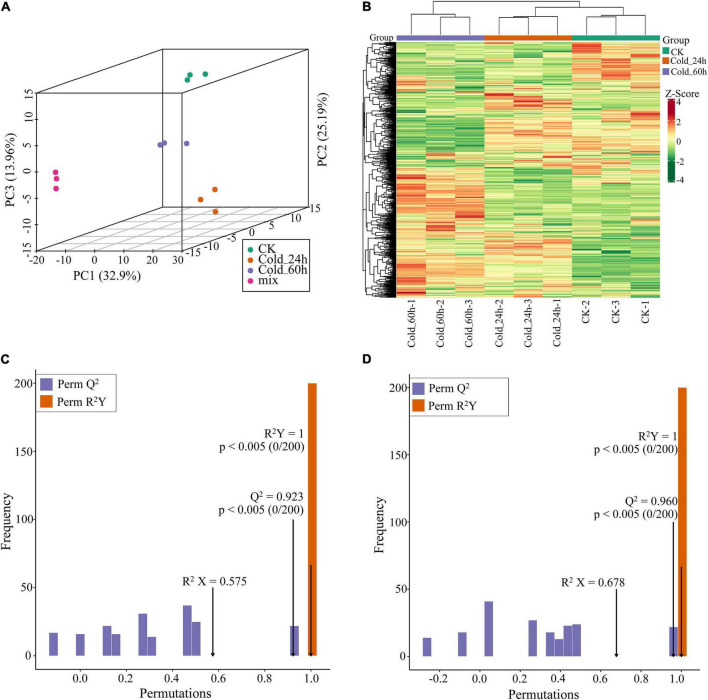
Multivariate statistical analysis of metabolites in the Antarctic moss *Pohlia nutans*. **(A)** Principal clustering analysis (PCA). **(B)** Hierarchical clustering analysis (HCA). **(C,D)** The OPLS-DA model supervised and calculated the variables responsible for differences between groups. R^2^X and R^2^Y indicate the interpretation rate of X and Y matrix, respectively. Q^2^Y represents the prediction ability of the model. A value closer to 1 means that the model is more stable and reliable. In addition, Q^2^Y > 0.5 can be regarded as an effective model, and Q^2^Y > 0.9 is an excellent model. C, Cold_24 h vs. CK. D, Cold_60 h vs. CK.

To uncover the potential mechanisms of *P. nutans* adapted to cold stress, the metabolites were detected using the UPLC-MS/MS method. Three principal components of PCA score plot (PC1, PC2, and PC3) were calculated to be 32.90%, 25.19%, and 13.96%, respectively. Four groups (i.e., CK, Cold_24 h, Cold_60 h, and mix) were clearly separated, and three biological replicates of each group were clustered together (Figure 2A). Therefore, PCA score plot showed that the experiments were reproducible and reliable. In addition, samples were obviously separated into three groups on the heatmap (Figure 2B), suggesting that there were significant differences in the classes and quantities of metabolites among three groups. In addition, the supervised model of OPLS-DA compared metabolite contents and identified the variables responsible for differences between groups. Then, the differences were calculated by OPLS-DA model between Cold_24 h and CK (R^2^X = 0.575, R^2^Y = 1, Q^2^ = 0.923), and between Cold_60 h and CK (R^2^X = 0.678, R^2^Y = 1, Q^2^ = 0.960) (Figure 2C). The Q^2^ values of two comparisons were both larger than 0.9, indicating that these models were stable and significant differences were occurred in metabolic phenotypes after cold stress (Figure 2D). These results showed that cold stress strongly changed the metabolite profiles of the Antarctic moss *P. nutans*.

A total of 559 metabolites were detected which included 78 Free fatty acids, 77 Amino acids and derivatives, 77 Phenolic acids, 54 Organic acids, 51 Saccharides and Alcohols, 43 Nucleotides and derivatives, 30 Lysophosphatidylethanolamine (LPC), 24 Lysophosphatidylcholine (LPE), 19 Alkaloids, 17 Glycerol ester, 16 Flavonols, 11 Flavones, 11 Phenolamine, 11 Vitamin, six Plumerane, five Flavanones, five Triterpene, four Flavanols, three Coumarins, two Piperidine alkaloids, two Stilbene, one Chalcones, one Monoterpenoids, one Phosphatidylcholine (PC), one Sesquiterpenoids, one Sphingolipids, and eight Others (Supplementary Table 2). Then, flavonoid biosynthesis pathway products including chalcones, flavones, flavonols, flavanonols, flavanones, and flavanols accounted for 6.62% of the total compounds. Of them, aromadendrin, dihydroquercetin, eriodictyol, luteolin, and kaempferol, the key intermediate metabolites of flavonoid synthesis pathway, were detected in the Antarctic moss *P. nutans*. Nevertheless, flavonoids in *P. nutans* mainly presented as the form of *O-*linked glycosylation modification, such as apigenin-7,4’-di-*O-*glucoside, luteolin-4’-*O-*glucoside, luteolin-7-*O-*gentiobioside, quercetin-3-*O-*glucoside, and quercetin-4’-*O-*glucoside (Supplementary Table 2).

**FIGURE 3:**
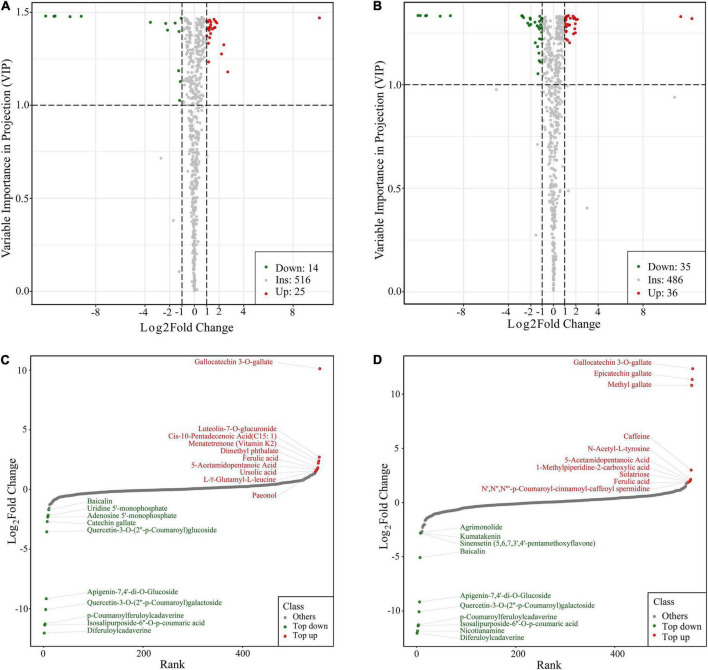
Identification of differently accumulated metabolites in the Antarctic moss *Pohlia nutans* under cold stress. **(A,B)** The volcano plot showing the contents of metabolites and the statistical significance. **(A)**, Cold_24 h vs. CK. **(B)**, Cold_60 h vs. CK. Each point represents a metabolite. Horizontal ordinate indicates the fold change of metabolites between two groups, while VIP value represents significant difference in statistical analysis. **(C,D)** The fold change of the top 20 different metabolites between two groups. **(C)**, Cold_24 h vs. CK. **(D)**, Cold_60 h vs. CK.

Using thresholds of | log_2_Fold Change| ≥ 1 and VIP (variable importance in project, VIP) ≥ 1, 39 significantly changed metabolites (SCMs) were detected between Cold_24 h and CK (25 upregulated, 14 downregulated) (Figure 3A and Supplementary Table 3), and 71 between Cold_60 h and CK (36 upregulated, and 35 downregulated) (Figure 3B and Supplementary Table 4). In particular, most of these differential metabolites were secondary metabolites, including alkaloids, flavonoids, terpenoids, and phenolic acids. The top 20 different metabolites were shown between two groups according to the values of log_2_Fold change, respectively. In the comparison of Cold_24 h vs. CK, gallocatechin 3-*O-*gallate belonged to flavanols, was the most significantly accumulated metabolite with log_2_(Fold Change) of 10.12 (Figure 2C). Luteolin-7-*O-*glucuronide, a kind of flavones, was the second significantly accumulated metabolite with log_2_(Fold change) of 2.7. In the comparison of Cold_60 h vs. CK, gallocatechin 3-*O-*gallate, epicatechin gallate, and methyl gallate were the most accumulated metabolites (Figure 2D). Moreover, the downregulated compounds were similar in both Cold_24 h vs. CK and Cold_60 h vs. CK comparison groups, respectively. For example, apigenin-7,4’-di-*O-*glucoside, quercetin-3-*O-*(2”-p-coumaroyl)galactoside, p-coumaroylferuloylcadaverine, isosalipurposide-6”-*O-*p-coumaric acid, and diferuloylcadaverine were the most significantly decreased metabolites after cold stress in these two comparison groups (Figures 2C,D).

**FIGURE 4:**
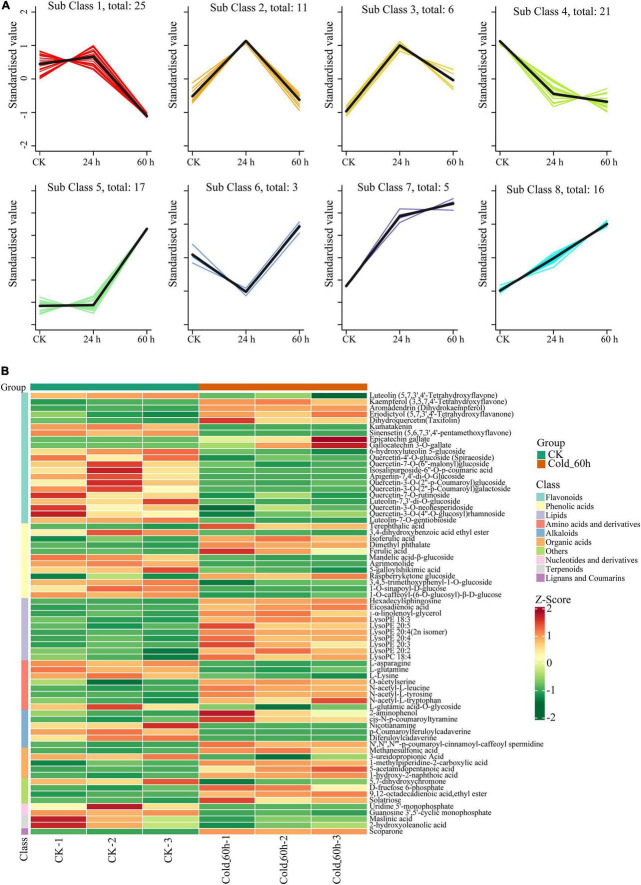
Cluster analysis of significantly different metabolites in *Pohlia nutans* after cold stress. **(A)** k-means clustering algorithm analysis of metabolites. **(B)** Heatmap of significantly different metabolites sorted by metabolite classes in *Pohlia nutans* after 60 h of cold stress.

Furthermore, eight different change tendencies of metabolites were found in *P. nutans* after cold stress by cluster analysis (Figure 4A). Flavonoids accounted for 29.58% of the total significantly different metabolites in Cold_60 h vs. CK (Figure 4B). Among the significantly different flavonoids with a total of 21 compounds, 12 compounds were modified by glycosylation to different degrees. Interestingly, except gallocatechin 3-*O-*gallate, most of *O-*linked glycosylated flavonoids such as quercetin-4’-*O-*glucoside, luteolin-7,3’-di-*O-*glucoside, apigenin-7,4’-di-*O-*glucoside, and quercetin-7-*O-*rutinoside were all gathered in Class 1 and Class 4, showing that their content was largely decreased after cold stress (Figure 4A and Supplementary Table 5). The contents of three lipids compounds [i.e., *Cis-*10-Pentadecenoic Acid (C15: 1), LysoPC 20:0, and LysoPE 15:0(2n isomer)] were increased in Cold_24 h groups and then recovered to the original level. Particularly, all unsaturated lipids compounds were clustered in Class 8 and their contents were continuously increased from 0 h to 60 h after cold stress. The contents of other lipids compounds also showed an overall increase trend. However, compounds of phenolic acids, amino acids and derivatives, and alkaloids demonstrated a diverse variation tendency (Figure 4A and Supplementary Table 5). Therefore, we proposed that the changes in content of these metabolites might contribute to increased membrane fluidity and protective action against reactive oxygen species (ROS) under cold stress.

### Transcriptome sequencing and differentially expressed genes annotation of *Pohlia nutans* under cold stress

We utilized the quantitative transcriptome sequencing strategy to reveal the transcriptional profiles of the Antarctic moss *P. nutans* under cold stress. The cDNA fragments were ligated with UMI barcodes to detect the duplicate reads caused by PCR amplification, and then sequenced on the Illumina novaseq 6000 Platform. We summarized the data output quality of quantitative transcriptome sequencing, including the amounts of raw reads, clean reads and UMI reads, and the ratio of Q20/Q30, GC content, UMI clean reads and deduplicate mapped UMI reads (Supplementary Table 6). After quality trimming, a total of 78.17 Gb clean reads was obtained from 12 samples, and UMI labeled reads accounted for 95.93%–96.39% of clean reads. For all libraries, the Q30 percentage was greater than 91.00%, and the average GC content was 51.61%. The number of UMI reads after deduplication accounted for 73.08%–76.72% of the number of UMI reads on the reference genome. The average mapping rate of UMI clean reads to *P. nutans* genome was 96.75% (Supplementary Table 7). After removing the non-natural duplicated reads, the correlation of gene expression levels between samples were analyzed by Pearson’s correlation test. The square of the Pearson’s correlation coefficient (R^2^) between samples within each group was greater than 0.99, indicating that subsequent differential gene analysis was more reliable (Supplementary Figure 1A). To discover overall differences in gene expression levels, we made a violin plot of the FPKM distribution under different cold treatments. FPKM values of the control group was higher than those of the cold treatment groups, indicating that the cold treatment inhibited gene expression (Supplementary Figure 1B). Above all, these results demonstrate an effective cold treatment of *P. nutans* gametophytes and a high-quality bioinformatics analysis for our RNA-sequencing results.

**FIGURE 5:**
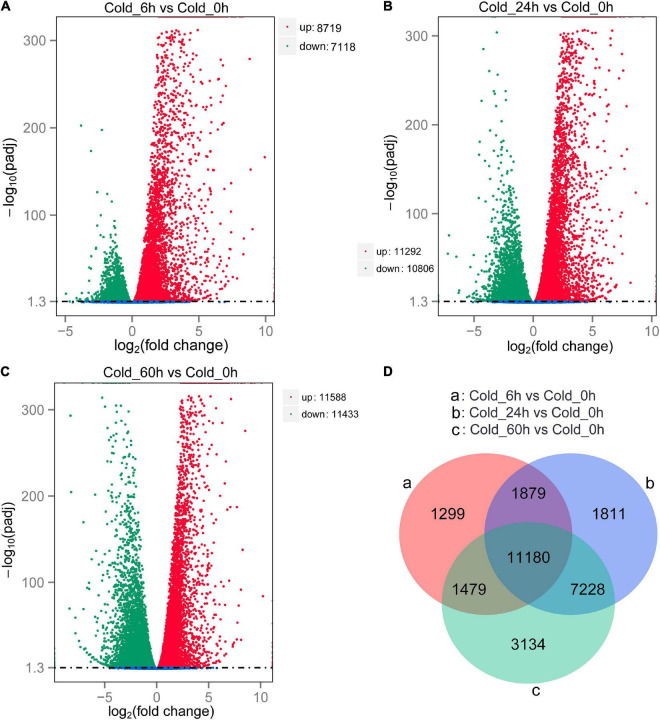
Differentially expressed genes screening and Venn diagram analysis **(A–C)**. The volcano plot showing the DEGs between cold stress group and control group. The X-axis indicates fold change of gene expression (threshold, | log_2_(Treat/Control)| > 0), while the Y-axis means the statistically significant level (threshold, log10(padj) > 1.3). **(D)** Venn diagram shows the DGEs distribution among different compare groups.

We further evaluated the global gene expression profiles by investigating the DEGs associated with cold response in *P. nutans*. A total of 15,837 DEGs (8,719 up- and 7,118 down-regulated) in Cold_6 h vs Cold_0 h, 22,098 DEGs (11,292 up- and 10,806 down-regulated) in Cold_24 h vs Cold_0 h, and 23,021 DEGs (11,588 up- and 11,433 down-regulated) in Cold_60 h vs Cold_0 h were identified, respectively (Figures 5A–C). Venn diagram showed that 11,180 DEGs were shared among Cold_6 h vs Cold_0 h, Cold_24 h vs Cold_0 h, and Cold_60 h vs Cold_0 h (Figure 5D). The representative DEGs in different gene families were selected and calculated (Table 1). According to their function, the DEGs were further classified into different classes including Ca^2+^ signaling, ABA signaling, Jasmonate signaling, Auxin signaling, Gibberellin signaling, Fatty acids biosynthesis, Flavonoid biosynthesis, Antioxidant enzymes, Transcription factors, and other gene families, as well as several novel gene families related to cold stress. We proposed that the global transcriptional regulation of stress-related genes might contribute to fine-tuning the cold responses in the Antarctic moss *P. nutans*.

**TABLE 1:** Representative cold stress-related genes of the Antarctic moss *Pohlia nutans.* Data were summarized from the Supplementary Tables 8–10.

Function or signaling pathway	Gene family	Cold_6 h vs Cold_0 h		Cold_24 h vs Cold_0 h		Cold_60 h vs Cold_0 h	
		Log_2_Fold Change (Treat/Control)	Ratio (up-regulated genes/total genes)	Log_2_Fold Change (Treat/Control)	Ratio (up-regulated genes/total genes)	Log_2_Fold Change (Treat/Control)	Ratio (up-regulated genes/total genes)
Ca^2+^ signaling	Calmodulin	0.25∼6.91	10/10	0.18∼5.04	10/10	−0.47∼3.66	4/5
	Calmodulin-binding protein 60	0.99∼2.10	4/4	0.88∼1.52	4/4	−0.42∼0.63	1/2
	Probable calcium-binding protein CML	1.46∼7.42	23/23	−0.41∼5.06	19/25	−2.76∼3.35	15/22
	Calmodulin-binding receptor-like cytoplasmic kinase	−1.17∼1.83	3/5	−1.44∼1.35	6/7	−2.32∼1.10	5/7
	Calcium-activated potassium channel	−0.80∼2.37	4/6	−2.64∼2.67	4/8	−2.45∼1.30	2/7
	Calcium-dependent protein kinase	−0.80∼2.44	9/12	−2.09∼1.91	9/18	−2.90∼0.33	1/15
	Calcium-transporting ATPase	0.39∼2.81	15/15	−0.71∼4.54	16/17	−1.46∼4.67	13/14
	Sodium/calcium exchanger NCL1	1.66∼3.05	4/4	−0.83∼2.26	4/6	−1.17∼1.13	1/4
	Synaptotagmin	0.45∼3.52	9/9	−1.05∼3.28	8/14	−2.15–2.72	8/15
ABA signaling	9-*cis-*epoxycarotenoid dioxygenase, NCED3	0.26∼5.19	6/6	−0.45∼4.97	4/5	−1.34∼4.30	3/5
	Abscisic acid receptor, PYL4/8	−0.64∼2.35	2/6	−1.50∼1.90	3/7	−1.54∼0.70	3/8
	Abscisic acid-insensitive 5, ABI5	2.33∼2.39	2/2	2.09∼2.18	2/2	1.36∼1.57	2/2
	Abscisic acid and environmental stress-inducible protein, TAS14	2.63	1/1	2.12	1/1	0.43	1/1
	Probable protein phosphatase 2C	−1.34∼3.87	30/35	−2.36∼3.56	36/44	−3.00∼2.59	34/46
Jasmonate signaling	12-oxophytodienoate reductase 1/7/11	−0.13∼4.91	16/17	−2.24∼4.67	11/16	−4.35∼3.85	9/19
	Putative 12-oxophytodienoate reductase	−0.13∼4.23	9/10	−1.83 4.67	6/8	−3.61∼3.85	6/11
	Protein NINJA homolog	2.72∼2.90	2/2	1.77∼2.05	2/2	1.25∼1.32	2/2
	Protein TIFY	0.39∼4.20	10/10	0.50∼1.51	9/9	−0.81∼0.93	4/5
Auxin signaling	Auxin response factor, ARFs	−0.91∼0.98	12/15	−1.14∼1.10	9/15	−1.60∼0.51	4/17
	Auxin-responsive protein, SAURs	−1.63∼2.46	2/6	−2.35∼1.82	3/7	−3.83∼0.39	1/6
	Auxin-induced protein 5NG4/6B	−1.23∼1.96	3/6	−1.40∼2.54	6/8	−2.32∼1.78	1/6
	Transport inhibitor response 1-like protein	0.69∼1.10	2/2	1.25∼2.34	2/2	0.73∼0.98	2/2
Gibberellin signaling	Gibberellin 20 oxidase	−2.12∼5.12	11/15	−2.20∼3.54	12/19	−3.86∼4.71	10/17
	Gibberellin 2-beta-dioxygenase (GA-deactivating enzyme)	−0.60∼1.96	1/3	−1.77∼−1.23	0/3	−4.65∼−0.40	6/6
	DELLA protein, GAI1	−2.71∼2.48	2/9	−2.74∼2.13	7/11	−2.77∼0.54	2/10
Fatty acids	3-ketoacyl-CoA synthase	−0.66∼2.94	4/8	−0.94∼1.85	8/13	−1.38∼1.37	6/12
	Acyl-lipid (8−3)-desaturase	0.43∼3.64	6/6	1.21∼4.97	7/7	1.83∼3.71	6/6
	Fatty acid desaturase 4	2.72∼4.06	2/2	3.51∼4.33	2/2	3.28∼4.11	2/2
	Omega−3/6 fatty acid desaturase	−0.25∼0.87	3/4	−0.77∼2.24	3/4	0.24∼2.31	6/6
	Elongation of very long chain fatty acids protein	1.98∼2.25	2/2	−0.68∼3.33	2/3	−0.25∼2.54	3/4
	Enoyl-[acyl-carrier-protein] reductase	−0.56∼2.46	8/9	−1.68∼4.70	9/20	−3.37∼4.44	13/20
	Sn1-specific diacylglycerol lipase	−0.73∼4.37	12/13	0.67∼4.59	10/10	−0.77∼3.57	10/12
	Putative lipid phosphate phosphatase 3	4.09∼5.80	2/2	4.03∼5.35	2/2	2.62∼4.31	2/2
	Chalcone synthase	−0.64∼3.47	7/10	−1.67∼5.46	19/20	−2.47∼4.58	17/20
	Cinnamoyl-CoA reductase	−0.37∼1.18	8/9	−1.70∼2.64	8/13	−2.22∼4.60	6/13
	Flavonoid 3’-monooxygenase	−1.46∼0.55	3/7	−3.98∼4.59	10/16	−4.71∼4.43	8/13
	Flavonoid 3’,5’-hydroxylase	−1.15∼1.44	5/13	−6.30∼2.61	6/20	−3.13∼2.11	3/21
	GLABRA2 expression modulator	5.63	1/1	4.26	1/1	4.13	1/1
Antioxidant enzymes	Peroxisomal catalase	2.44	1/1	2.53∼2.98	2/2	1.74∼2.59	2/2
	Catalase	0.15∼3.23	3/3	−0.99∼4.53	3/5	−2.13∼4.48	3/5
	Glutaredoxin	0.22∼3.14	7/7	0.87∼3.54	8/8	0.54∼2.89	7/7
	Glutathione S-transferase	−1.64∼3.00	27/40	−2.31∼3.01	25/50	−6.98∼4.05	21/48
	Glutathione synthetase	1.05	1/1	2.09	1/1	0.60∼2.62	2/2
	Endoplasmic reticulum oxidoreductin	1.66∼3.42	4/4	0.75∼3.51	3/3	0.90∼2.63	3/3
	Cytochrome c	2.10∼2.43	2/2	3.17∼3.24	2/2	2.90∼2.94	2/2
	Mitochondrial uncoupling protein	−0.76∼5.44	11/13	−1.44∼4.93	10/12	−1.84∼5.08	8/10
	Protein DETOXIFICATION	−2.25∼3.27	18/24	−2.86∼4.34	23/31	−3.76∼4.24	20/31
Transcription factors	Ethylene-responsive transcription factor	−4.17∼8.81	68/83	−2.80∼6.98	59/73	−4.04∼5.16	36/58
	Dehydration-responsive element-binding protein	2.77∼7.36	15/15	−2.97∼6.24	13/15	−2.87∼5.74	12/15
	B-box zinc finger protein	0.53∼5.47	10/10	−3.14∼2.77	9/11	−2.55∼3.26	8/10
	Probable WRKY transcription factor	−1.97∼6.59	15/17	−2.16∼5.56	13/15	−2.77∼4.58	10/14
	Transcription factor MYB	−2.35∼4.74	13/22	−1.58∼4.58	20/23	−1.74∼1.57	9/19
Other gene families	Beta-carotene 3-hydroxylase	1.15∼6.85	4/4	2.17∼5.08	4/4	1.38∼5.52	4/4
	Beta-amylase	−1.15∼4.22	6/8	−2.76∼5.15	6/8	−2.84∼3.69	6/8
	Desiccation stress protein	3.33∼6.59	10/10	2.76∼6.78	10/10	1.12∼6.39	9/9
	Senescence/dehydration-associated protein	1.00∼8.37	16/16	−0.32∼6.56	16/17	−1.00∼6.17	2/16
	E3 ubiquitin-protein ligase COP1	0.72∼3.02	8/8	0.35∼3.24	6/6	−1.27∼2.54	5/6
	Early light-induced protein	3.48∼7.42	8/8	7.35∼7.71	8/8	7.51∼8.52	8/8
	Hydrophobic protein RCI2A	1.99∼4.10	3/3	2.88∼4.10	3/3	2.55∼2.81	3/3
	Late embryogenesis abundant protein	3.60∼6.25	14/14	2.55∼6.89	13/13	1.00∼6.91	12/12
	Low temperature-induced protein	0.97∼4.04	5/5	1.74∼5.41	5/5	0.73∼5.04	5/5
	Probable plastidic glucose transporter	2.01∼4.16	5/5	1.69∼3.28	5/5	0.69∼2.80	5/5
	Probable vacuolar amino acid transporter	2.10∼4.47	3/3	−0.90∼4.65	4/5	−1.20∼5.97	4/5
	Protein SRC2 homolog	−0.52∼7.07	8/11	−1.68∼5.55	8/11	−2.95∼4.41	8/14
	Translocator protein	1.48 5.99	8/8	−0.41∼5.98	8/9	−0.40∼5.13	9/10
	Low molecular mass early light-inducible protein	4.11∼5.51	3/3	4.11∼6.29	3/3	5.24∼7.16	3/3
	High molecular mass early light-inducible protein	1.47∼7.54	14/14	−2.24∼9.57	15/17	−2.25∼10.22	13/16
	Integrin-linked protein kinase	−1.17∼3.97	11/13	−2.69∼2.32	8/12	−2.24∼1.29	6/12
Novel genes related to cold stress	Membrane protein PM19L	−0.47∼6.36	10/11	−1.75∼6.23	10/12	−2.40∼5.02	9/14
	Transmembrane protein 53/205	0.33∼6.67	11/11	0.56∼6.50	11/11	0.63∼4.90	11/11
	Acyltransferase-like protein	−0.40∼1.53	5/6	0.58∼3.72	5/5	−1.50∼3.62	5/6
	Polyprenol reductase	2.87∼3.67	2/2	3.87∼2.66	2/2	2.12∼3.26	2/2
	Ferritin−3	1.37∼2.22	4/4	2.21∼5.42	4/4	1.69∼3.84	4/4
	Vacuolar iron transporter	3.75∼4.01	3/3	−0.82∼7.36	3/5	2.79∼5.04	3/3
	Monosaccharide-sensing protein	1.98∼5.58	4/4	2.07∼5.40	4/4	1.52∼4.93	4/4
	Nitrate reductase	3.95∼4.15	2/2	1.41∼2.00	2/2	1.27∼1.36	2/2
	Protein MOTHER of FT and TFL1	1.94∼4.81	4/4	3.20∼4.81	4/4	2.41∼4.42	4/4
	Protein DOG1-like 3	3.57∼6.61	4/4	2.48∼4.30	4/4	2.25∼3.37	4/4
	Phospholipase A2-alpha	3.88∼6.42	2/2	5.12∼7.35	2/2	5.41∼8.18	2/2

### Differentially expressed genes related to fatty acid pathway

**FIGURE 6:**
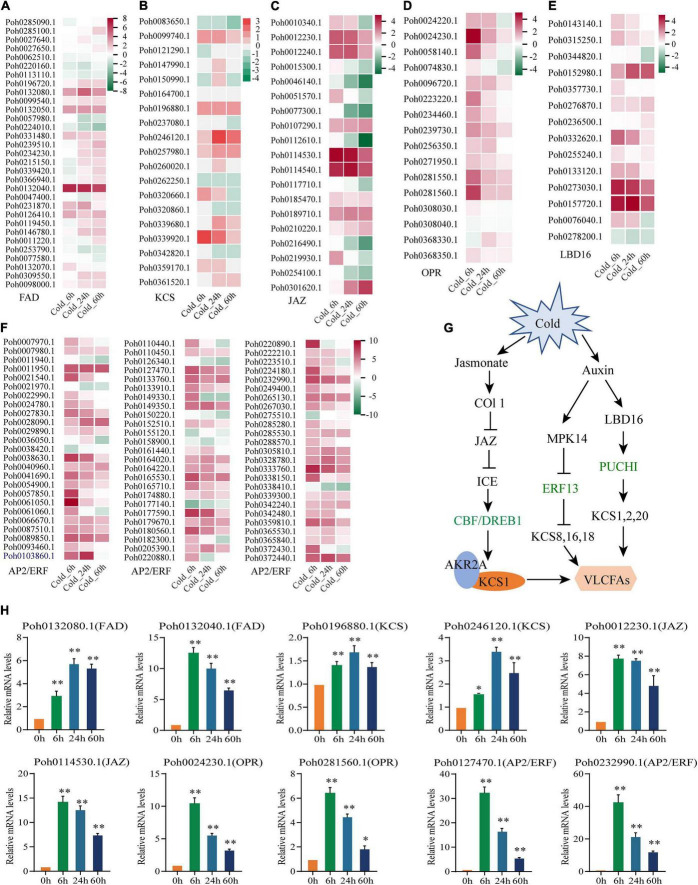
Genes involved in the biosynthesis pathways of very-long-chain fatty acids were upregulated under cold stress. **(A)** fatty acid desaturases (*FAD*), **(B)** β-keto-acyl-CoA synthase (*KCS*), **(C)** Jasmonate-Zim Domain (JAZ) proteins (*JAZ*), **(D)** 12-oxophytodienoate reductase (*OPR*), **(E)** LATERAL ORGAN BORDER DOMAIN 16 (*LBD16*), and **(F)** AP2/ERF transcription factor. Heatmaps show the gene expression profile at different time points of cold stress, which were markedly upregulated detected by transcriptome sequencing. As gene expression level increases, the color changes from green (low accumulation) to red (high accumulation). **(G)** A proposed model summarizing the main pathways for *KCS* gene expression and very-long-chain fatty acids biosynthesis under cold stress. **(H)** Several genes involved in the biosynthesis pathway of very-long-chain fatty acids were significantly upregulated under cold stress confirmed by qPCR analysis. Significant difference (*P < 0.05 and **P < 0.01).

Previously, we have found that several gene families were expanded which participated in biosynthesis of metabolites such as fatty acid elongation and alpha-Linolenic acid metabolism (Liu et al., 2022). Here, we found that the content of very-long-chain fatty acids (VLCFAs) increased significantly under cold stress in metabolome analysis. We will focus on the regulation mechanism of very long chain fatty acids (VLCFAs) biosynthesis. In the *P. nutans* genome, gene families encoding fatty acid desaturases were expanded, including FAD3 (omega-3 fatty acid desaturase), FAD6 (omega-6 fatty acid desaturase), and cytochrome b5 fatty acid desaturase (Supplementary Figure 2A). Under cold stress, these genes showed marked upregulation (Figure 6A). The gene family of CoA synthase (KCS), the rate-limiting enzyme responsible for the synthesis of VLCFAs, was found to have been expanded in *P. nutans*. There were 19 of KCS homologs in the *P. nutans* genome, 13 in the *C. purpureus* genome, 13 in the *P. patens* genome, four in the *M. polymorpha* genome, and four in the *A. angustus* genome (Supplementary Figure 2B). In addition, eight of the KCS genes were significantly upregulated under cold stress (Figure 6B). Jasmonates modulate the ICE-CBF/DREB1 cascade to play an essential role in regulating cold stress. Jasmonate ZIM-domain (JAZ) proteins are transcriptional repressors that function as negative regulators of diverse jasmonic acid (JA) responses. The 12-oxo-phytodienoic acid reductases (OPRs) are important enzymes that catalyze the reduction of converting linolenic acid to JA. We found that the gene families of JAZ protein and OPR enzyme were expended in *P. nutans* when compared with other four bryophytes (Supplementary Figures 2C,D). Under cold stress, approximately half of the JAZ genes were significantly upregulated, while most of the OPR genes were markedly upregulated (Figures 6C,D). According to recent research, auxin signaling is tightly correlated to cold stress-induced changes in plant development. LBD16 (LATERAL ORGAN BORDER DOMAIN 16) is one of the major regulatory molecules involved in the auxin signal pathway. A number of LBD16 homologous genes were up-regulated in *P. nutans* under cold stress (Figure 6E). Notably, CBF/DREB1, ERF13, and PUCHI are all the AP2/ERF transcription factors which were induced by jasmonate, auxin, and ethylene, but independent of ABA signal pathway. We found that most of the AP2/ERF transcription factors were significantly upregulated under cold stress (Figure 6F). Therefore, we suggest that AP2/ERF transcription factors play critical roles in orchestrating the crosstalk between the jasmonate and auxin signaling pathways in moss acclimating to cold stress. We summarized a proposed model of the main pathways for regulating the KCS gene expression and very-long-chain fatty acids biosynthesis under cold stress (Figure 6G; Hu et al., 2013; Chen et al., 2021; Guyomarc’h et al., 2021). In addition, we confirmed the up-regulated profiles of several selected genes under cold stress by qPCR analysis, including *FAD* (Poh0132080.1 and Poh0132040.1), *KCS* (Poh0196880.1 and Poh0246120.1), *JAZ* (Poh0012230.1 and Poh0114530.1), *OPR* (Poh0024230.1 and Poh0281560.1), and *AP2/ERF* (Poh0127470.1 and Poh0232990.1) (Figure 6H).

### Integrated metabolic and transcriptional analysis reveals the role of flavonoid pathway in cold stress

**FIGURE 7:**
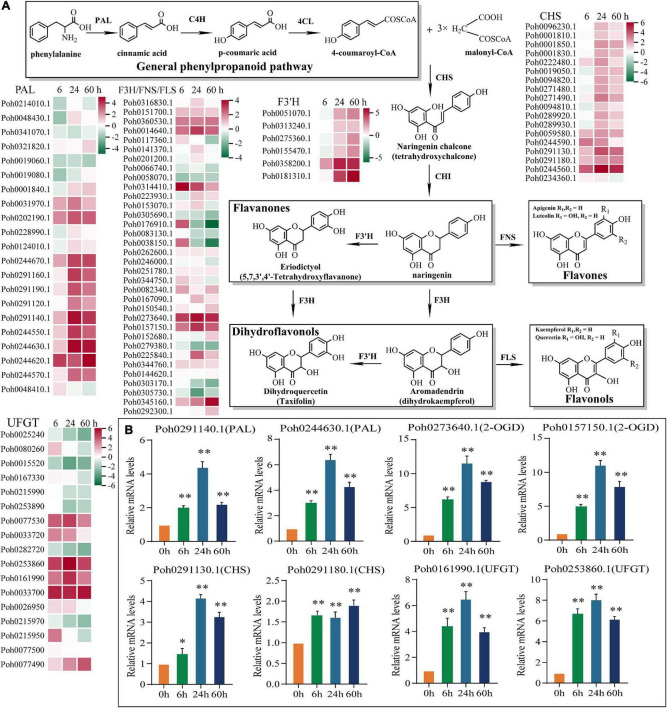
Genes involved in the biosynthesis pathways of flavonoids were upregulated under cold stress. **(A)** Proposed biosynthetic pathway of flavonoid synthesis in *Pohlia nutans*. Gene expression profile at different time points of cold stress. As gene expression level increases, the color changes from green (low accumulation) to red (high accumulation). Phenylalanine ammonia-lyase (*PAL*), cinnamate-4-hydroxylase (*C4H*), 4-coumarate CoA ligase 4 (*4CL*), chalcone synthase (*CHS*), chalcone isomerase (*CHI*), flavanone 3-hydroxylase (*F3H*), flavonol synthase (*FLS*), flavone synthase (*FNS*), UDP-flavonoid glucosyltransferase (*UFGT*). **(B)** Several flavonoid biosynthesis enzyme genes were markedly upregulated under cold stress detected by qPCR analysis. Significant difference (*P < 0.05 and **P < 0.01).

To gain further insights into flavonoid biosynthesis pathway, HMMER program and BLASTP alignment were performed to identify the key enzymes, including PAL, CHS, CHI, F3′H, and 2-OGD protein family (i.e., FNS, F3H, and FLS). Using data from the widely targeted metabonomic analysis, we found that *P. nutans* synthesizes flavones (Apigenin and Luteolin), flavanones (Eriodictyol and Naringenin), flavonols (Kaempferol and Quercetin), and dihydroflavonols (Aromadendrin and dihydroquercetin). By referring to previously published results from other higher plants, we outlined a model for flavonoid biosynthesis in *P. nutans* (Figure 7A). It was inevitable that there was a correlation between flavonoid contents and the expression levels of flavonoid-related genes. To comprehensively reveal the genes involved in the flavonoid biosynthesis pathways, we used heatmaps to show the expression levels of flavonoid synthase genes in transcriptome sequencing (Figure 7A). We identified the markedly up-regulated or down-regulated genes involved in flavonoid biosynthesis in *P. nutans*, including 22 *PAL*, 18 *CHS*, six *CHI*, 34 *2-OGD* (i.e., FNS, F3H, and FLS), and 17 UDP-glycose flavonoid glycosyltransferase (*UFGT*) that were retrieved from the data of gene differential expression analysis. Among them, we found that the expression levels of several genes which were closely related to flavonoid biosynthesis. In addition, we confirmed the up-regulated expression levels of these genes under cold stress by qPCR analysis, such as *PAL* (Poh0291140.1 and Poh0244630.1), *CHS* (Poh0291130.1 and Poh0291180.1), *2-OGD* (Poh0273640.1 and Poh0157150.1), and *UFGT* (Poh0161990.1 and Poh0253860.1) (Figure 7B). We therefore proposed that the up-regulated genes and accumulated metabolites in flavonoid biosynthesis pathway might contribute to enhancing plant resistance to cold stress in *P. nutans*.

## Discussion

In Antarctic regions, terrestrial plants are exposed to harsh conditions such as cold, drought, strong winds and high UV irradiances (Convey and Peck, 2019; Perera-Castro et al., 2021). These plants survive and propagate some of the harshest environments on earth, which have recently been aggravated by climate change and ozone depletion. Antarctic terrestrial ecosystem therefore offers a unique opportunity to disclose organism-environment interactions (Block et al., 2009; Convey and Peck, 2019; Bertini et al., 2021; Cannone et al., 2022). The Antarctic moss *P. nutans* grows abundantly in Fildes Peninsula and Victoria Land, when the snow melts and the summer water is plentiful (Skotnicki et al., 2002; Skotnicki et al., 2005). Typically, they appear as tiny and solitary colonies with short shoots (1–2 cm length). *P. nutans*, thriving on the geothermal ground of Victoria Land’s Mount Rittmann, has low levels of genetic diversity and appears to have originated from a single immigration incident followed by vegetative development, mutation, and spread (Skotnicki et al., 2002). Previously, we demonstrated that a lectin receptor-like kinase (i.e., PnLecRLK1) from *P. nutans* functions as a membrane bound regulator that increases chilling stress tolerance possibly through stimulating the CBF signaling pathway (Liu et al., 2017). We also investigated the transcriptome profiles of *P. nutans* under multiple stresses like cold stress, high salt stress, and UV-B radiation (Liu et al., 2013; Li et al., 2019; Zhang et al., 2019). In the present study, using a newly assembled genome, along with metabolomic and transcriptomic data across a treatment series of cold stress, we proposed a regulating network for the metabolic pathways and candidate genes underlying the adaptations of *P. nutans* to the polar terrestrial environments.

Metabolomics is currently being utilized to qualitatively and quantitatively evaluate all metabolites to offer an advanced assessment technique of the cellular state. The HPLC-MS/MS-based metabolomics are widely used to profile metabolites involved in the physiological responses to environmental challenges in plants (Liu et al., 2021; Sun et al., 2021; Xiong et al., 2022). In the present study, we utilized a widely targeted metabolomics approach using the UPLC-MS/MS analytical platform to analyze the metabolite changes of *P. nutans* under cold stress (Figure 2). A total of 39 SCMs were detected in Cold_24 h vs. CK (25 upregulated, 14 down-regulated), and 71 SCMs were detected in Cold_60 h vs. CK (36 upregulated, 35 down-regulated) based on the thresholds of | log2Fold Change| ≥ 1 and VIP ≥ 1 (Figure 3). Previously, the metabolite profiling of the moss *Physcomitrella patens* reveals unique metabolic patterns between protonema and gametophores, however, only a few metabolites were identified by GC-MS analysis (Erxleben et al., 2012). Cold stress usually has adverse effects on membrane fluidity, ROS homeostasis, and energy metabolism, and photosynthesis (Shi et al., 2018; Ding and Yang, 2022). Upon cold stress, plants accumulate excessive ROS, including superoxide (O_2_^–^), hydrogen peroxide (H_2_O_2_), and hydroxyl radicals (OH^–^), which are toxic molecules causing oxidative stress (Adhikari et al., 2022). We investigated the ROS scavenging mechanisms of *P. nutans*. We found that several gallic acid-derived compounds (i.e., gallocatechin 3-*O-*gallate, epicatechin gallate, and methyl gallate) were the most accumulated metabolites under cold stress (Figure 4), which usually have strong antioxidant activities. For instance, epigallocatechin-3-gallate and epicatechin gallate generally obtains from green tea and functions as the powerful antioxidants, preventing oxidative stress from hydrogen peroxide- and radical-induced damages (Singh et al., 2011; He et al., 2018; Tang et al., 2021; Yu et al., 2021). Methyl gallate, methyl-3,4,5-trihydroxybenzoic acid, enhances antioxidant enzymes and protects a diversity of cells from oxidative stress through hydrogen peroxide- and radical-scavenging activities (Ng et al., 2018). It also decreases lipid peroxidation and prevents depletion of intracellular glutathione (Hsieh et al., 2004). Thus, it is reasonable to assume that these gallic acid-derived metabolites are associated with ROS scavenging and antioxidant activities in *P. nutans* under cold stress.

Flavonoids are widely distributed metabolites in land plants. During the evolution from aquatic to terrestrial, various kinds of flavonoids such as flavonols and anthocyanins emerged sequentially, facilitating adaption of land plants to the harsh terrestrial environment (Ni et al., 2019; Liu et al., 2021). However, little is known about flavonoid content and the molecular process of flavonoid biosynthesis in these basal land plants like bryophytes (Li et al., 2020). Flavonoids was the main compounds that accounted for 29.58% of the total significantly different metabolites in Cold_60 h vs. CK in *P. nutans* (Figure 4 and Supplementary Table 4). We also identified several significantly up-regulated or down-regulated family genes involved in flavonoid biosynthesis in *P. nutans* (Figure 7). Flavonoids are natural phenolic compounds with significant health benefits for both plants and humans. Eriodictyol, a flavonoid usually found in fruits and vegetables, has been reported to have strong anti-inflammatory, anti-apoptotic, and antioxidant properties (Wang et al., 2021a). The content of eriodictyol was significantly increased in *P. nutans* under cold treatment (Figure 4). Intriguingly, we found that most of *O-*linked glycosylated flavonoids among the detected DCMs were downregulated under cold stress (Figure 4). Glycosylation can change solubility, and stability, as well as influence cellular localization and biological activities of flavonoids, while deglycosylation represents an extremely important regulation way in flavonoids homeostasis (Le Roy et al., 2016). In most cases, the *O-*glycosylation of flavonoids reduced their antioxidant capacities which was determined by *in vitro* experiments using superoxide radical scavenging assays (Xiao et al., 2014). The accumulation of flavonoid glycosides likely constitutes a reserve pool of precursors which could be easily mobilized under unfavorable growth conditions (Le Roy et al., 2016; Johnson et al., 2021). Thus, the dynamic changes of flavonoid constituents under cold stress might facilitate mosses in resisting the extra ROS damages.

Alteration of cell membrane fluidity is considered to be an important survival strategy for plants under cold stress. This process largely relies on regulating the content of polyunsaturated fatty acids (PUFAs). We found that gene families encoding fatty acid desaturases were expanded in the *P. nutans* genome, including FAD3 (omega-3 fatty acid desaturase), FAD6 (omega-6 fatty acid desaturase), and cytochrome b5 fatty acid desaturase (Figure 4A). These genes were markedly upregulated under cold stress (Figure 6H). Very-long-chain fatty acids (VLCFAs) are exclusively component in several membrane lipids and crucial for membrane homeostasis (Batsale et al., 2021). We found that gene family of β-keto-acyl-CoA synthase (KCS), the rate-limiting enzyme for the synthesis of VLCFAs, were expanded in *P. nutans* (Figure 6H). In addition, three *KCS* were significantly upregulated under cold treatment. Notably, among significantly changed metabolites (SCMs), we found that the content of PUFAs were all increased under cold stress. These PUFAs were almost all VLCFAs, except hexadecylsphingosine with 16 carbons (Figure 4B).

Jasmonates include jasmonic acid (JA), methyl jasmonate, jasmonoyl-L-isoleucine (JA-Ile), and 12-oxo-phytodienoic acid (OPDA), which play critical roles in modulating plant resistance to insect assaults and pathogen infection, and various abiotic stresses (Aslam et al., 2021). Jasmonates regulate multiple transcription factors such as JAZ, MYB, AP2/ERF and bHLH to play an essential role in regulating metabolites and stress responses (Hu et al., 2013; Wasternack and Strnad, 2019). We found the gene families involved in jasmonate signaling were expanded in the *P. nutans* genome, such as JAZ protein and 12-oxophytodienoate reductase (identified by NADH: flavin oxidoreductase domain). Their gene expression levels were upregulated under cold stress (Figure 6H). In addition, we discovered that gene families encoding AP2/ERF transcription factors were expanded in *P. nutans*. AP2/ERF transcription factor was usually induced by jasmonate, auxin, and ethylene but independent of ABA signal pathway. C-repeat/dehydration-responsive element-binding factors (CBF/DREB) were AP2/ERF transcription factors and were well-known to function in cold-response pathways. We identified the cold stress-regulated AP2/ERF and putative CBF/DREB transcription factors (Figure 6F). Through JAZ proteins, jasmonic acid can boost plant resilience to cold stress by regulating the ICE-CBF/DREB1 cascade (Hu et al., 2013), while ICE-CBF functions as a critical upstream signal of ankyrin repeat-containing protein (AKR2A) and KCS1 to improve VLCFAs contents and chilling tolerance (Chen et al., 2021). 12-oxo-phytodienoic acid (OPDA) is a precursor of jasmonic acid (JA) biosynthesis. Interestingly, the moss *P. patens* contains OPDA but no JA or its amino acid conjugates, while OPDA is deemed to regulate a signaling pathway distinct from JA (Dave and Graham, 2012). The role of OPDA in plant response to cold stress has hardly been reported. Our findings indicate that OPDA signaling plays an important role in low terrestrial plants against cold stress.

The plant hormone auxin regulates virtually all aspects of plant growth and development under optimum condition, but the knowledge of the role of auxin in abiotic stresses is limiting. The emerging trend from the recent investigations indicated that the intracellular auxin signaling is tightly linked to cold stress-induced changes in plant development. For example, PUCHI and ERF13 are both AP2/ERF transcription factors that collectively orchestrate the biosynthetic process of VLCFAs *via* the transcriptional control of multiple KCS genes (Figure 6B; Guyomarc’h et al., 2021). Our findings showed that almost all the AP2/ERF transcription factors were markedly upregulated under cold stress (Figure 6F). LATERAL ORGAN BOUNDARIES-DOMAIN 16 (LBD16) is one of the major regulators involved in auxin signaling. The expression levels of four LBD16 homologous genes were increased in *P. nutans* under cold stress (Figure 6E). LBD16 is located upstream of PUCHI in auxin signaling. The sequential activating of transcription factors LBD16 and PUCHI will synergistically regulate lateral root initiation in *Arabidopsis thaliana* (Goh et al., 2019). Taken together, we suggested that AP2/ERF transcription factors might play a central role in orchestrating very long chain fatty acid biosynthesis, involved in plant development and the adaptation to abiotic stresses (Figure 6G).

Overall, we conducted high-quality transcriptome sequencing and analysis, as well as metabolic profiling. Meanwhile, we also found some special mechanisms, such as the significant increase of acetylated amino acids in *P. nutans* under cold stress. The integrated metabolome and transcriptome analysis provides a comprehensive landscapes of the Antarctic moss *P. nutans* in response to cold stress. These results imply that *P. nutans* has developed the complicated synergistic mechanisms to acclimate to life in extreme terrestrial habitats, including plant hormone signaling, flavonoid biosynthesis, fatty acid biosynthesis, and ROS scavenging system.

## Data availability statement

The original data presented in this study are publicly available. The whole genome sequence data were available in the National Genomics Data Center (NGDC, https://ngdc.cncb.ac.cn) under the BioProject number: PRJCA008231. The assembly and annotation data were deposited in Genome Warehouse (GWH) under the accession number: GWHBHNB00000000. The transcriptome sequencing data of *P. nutans* were also deposited in GSA under the accession number: CRA006556.

## Author contributions

SL and LZ conceived the original research and designed the experiments. SL performed the cold stress treatment, analyzed the data, and wrote the draft manuscript. TL conducted the quantitative RT-PCR analysis. SF measured the biochemical features. PZ performed the metabolite analysis. DY and BC performed the transcriptome analysis. ZZ and LZ supervised the experiments and revised the manuscript. All authors contributed to the article and approved the submitted version.

## Footnotes

1
www.r-project.org/

## Supplementary material

Supplementary Figure 1 Overall assessment of data quality and changes of RNA-sequencing. **(A)** The Pearson correlation coefficient (r^2^) was used to calculate the predictive accuracy of model comparison of RNA-sequencing data between different groups. **(B)** The violin diagram compares the gene expression levels under different experimental conditions. The violin map of each region corresponds to five statistics (top-down are the maximum, upper quartile, median, lower quartile, and minimum), and the width of each violin represents the number of genes under this expression.

Supplementary Figure 2 Gene families of fatty acids and very-long-chain fatty acids biosynthesis pathways were expanded in the *Pohlia nutans* when compared with other bryophyte genomes. **(A)** fatty acid desaturases, **(B)** β-keto-acyl-CoA synthase, **(C)** Jasmonate-Zim Domain (JAZ) proteins, **(D)** 12-oxophytodienoate reductase.
