# Supplementary figures and images for: Metabolic profiling and gene expression analyses provide insights into cold adaptation of an Antarctic moss Pohlia nutans
Source: Front Plant Sci. 2022 Sep 13;13:1006991. doi: 10.3389/fpls.2022.1006991 (PMC9514047; doi:10.3389/fpls.2022.1006991)

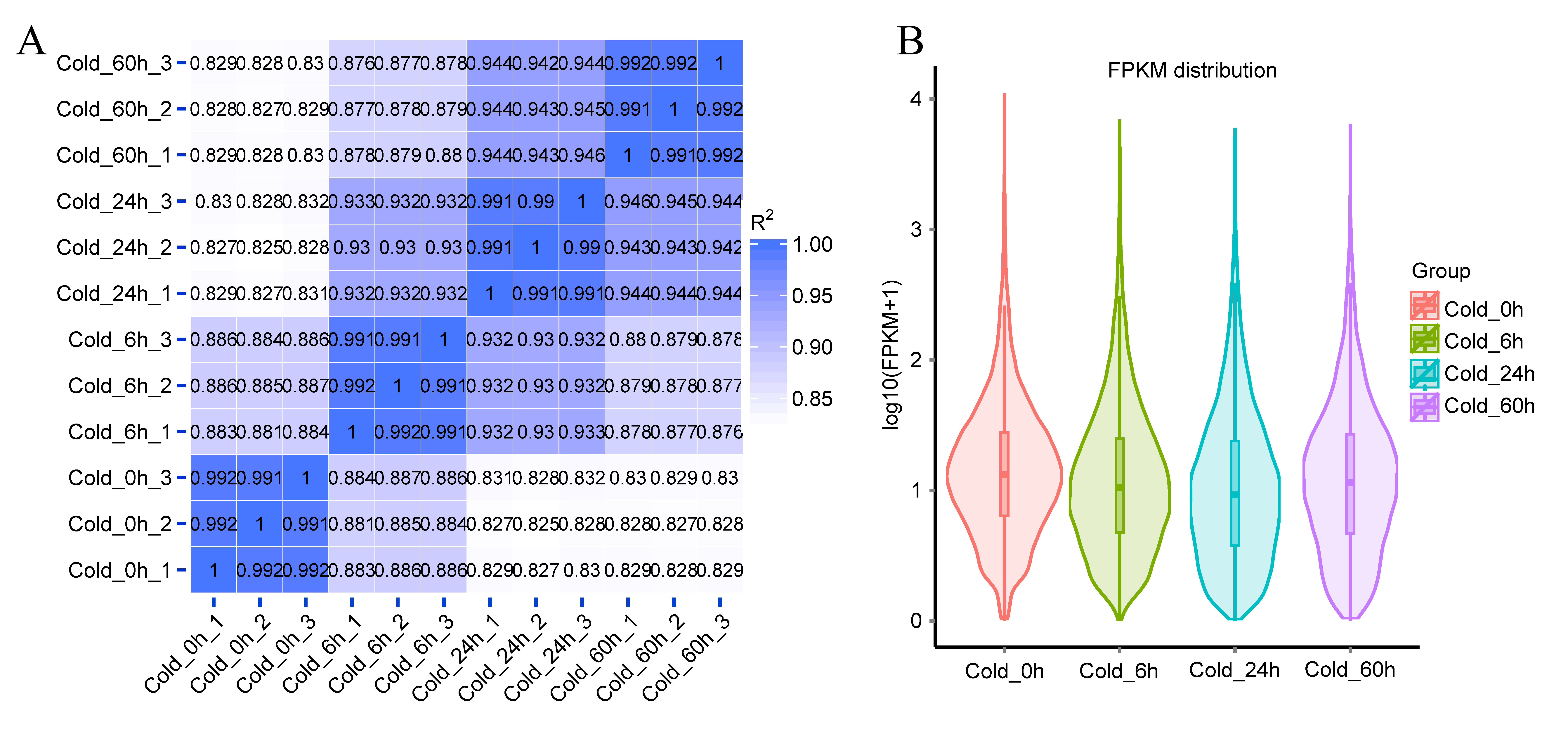

Supplement: Supplementary Figure 1 — Overall assessment of data quality and changes of RNA-sequencing. (A) The Pearson correlation coefficient (r2) was used to calculate the predictive accuracy of model comparison of RNA-sequencing data between different groups. (B) The violin diagram compares the gene expression levels under different experimental conditions. The violin map of each region corresponds to five statistics (top-down are the maximum, upper quartile, median, lower quartile, and minimum), and the width of each violin represents the number of genes under this expression. [file Image_1.TIFF]

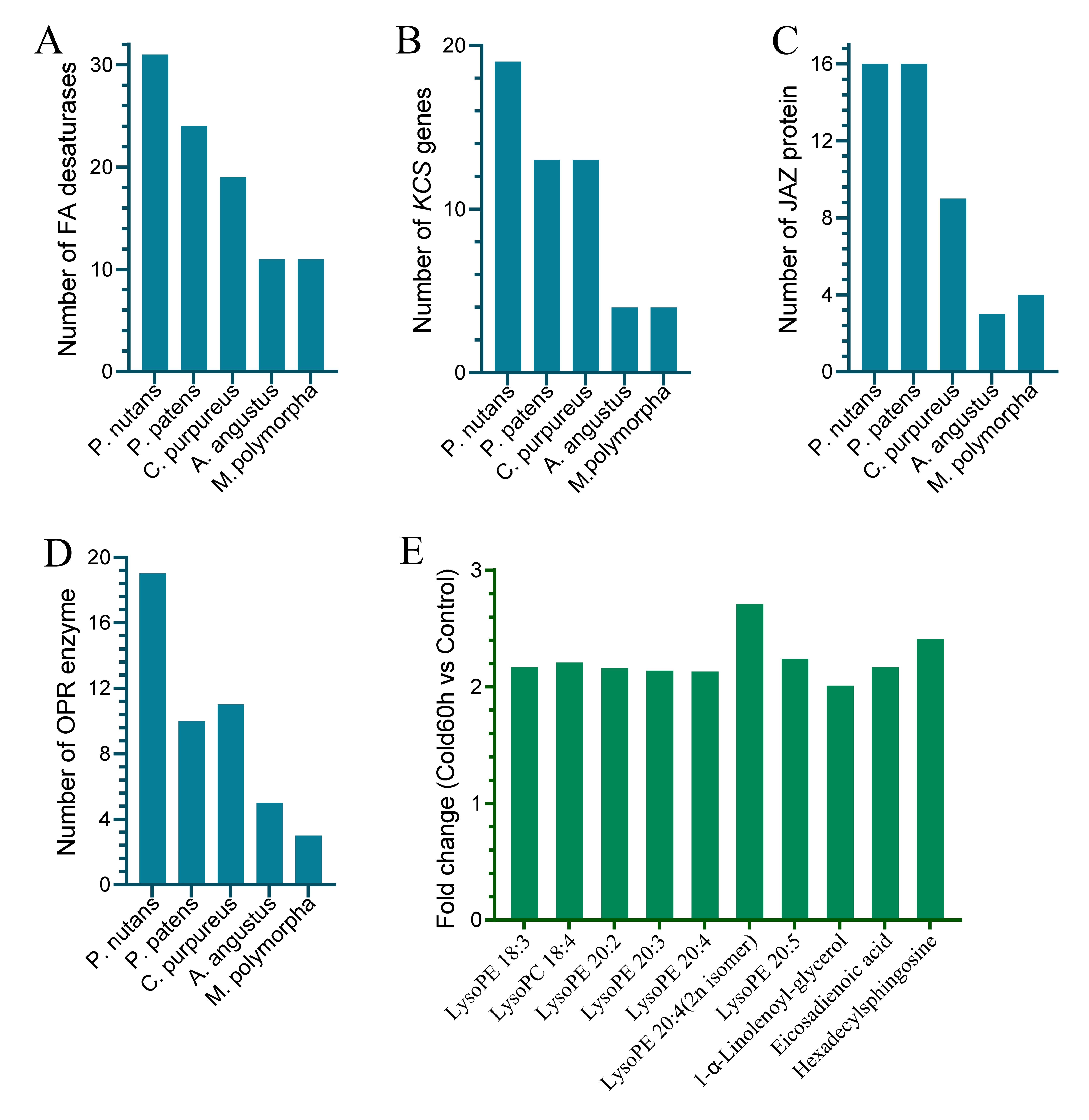

Supplement: Supplementary Figure 2 — Gene families of fatty acids and very-long-chain fatty acids biosynthesis pathways were expanded in the Pohlia nutans when compared with other bryophyte genomes. (A) fatty acid desaturases, (B) β-keto-acyl-CoA synthase, (C) Jasmonate-Zim Domain (JAZ) proteins, (D) 12-oxophytodienoate reductase. [file Image_2.TIFF]
