# Supplementary material for: Metabolic profiling and gene expression analyses provide insights into cold adaptation of an Antarctic moss Pohlia nutans
Source: Front Plant Sci. 2022 Sep 13;13:1006991. doi: 10.3389/fpls.2022.1006991 (PMC9514047; doi:10.3389/fpls.2022.1006991)
Supplement: Supplementary file 3 [file Table_1.DOCX]

**Supplementary Table 1** Primers used for quantitative real-time RT-PCR analysis.

| Gene ID | Gene symbol | Primer name | Primer sequence (5’-3’) | Annealing temperature (°C) | Product length (bp) |
| --- | --- | --- | --- | --- | --- |
| Poh0132080.1 | *FAD-1* | FAD01F | GGCGACTGGCGATTCTTGGT | 58.5 | 124 |
|  |  | FAD01R | TGTAAGGAACGACGGCATTGAT | 56.5 |  |
| Poh0132040.1 | *FAD-2* | FAD02F | GGCGACTGGCGATTCTTGGTT | 59.5 | 131 |
|  |  | FAD02R | GGGTAAATGTAAGGAACGACGGC | 58.1 |  |
| Poh0196880.1 | *KCS-1* | KCS01F | GGCAACACATCTTCCTCCTCCAT | 58.8 | 112 |
|  |  | KCS01R | ACTTAAACCCACTCCCAAACGCAAT | 60.0 |  |
| Poh0246120.1 | *KCS-2* | KCS02F | AGAAGCAAGTGGTGGCGTCGTA | 60.5 | 188 |
|  |  | KCS02R | CGTGATGAGATGCGTGATGTCGTC | 60.7 |  |
| Poh0012230.1 | *JAZ-1* | JAZ01F | GAGAATGGAGTGGCTGGCAATGG | 60.2 | 104 |
|  |  | JAZ01R | ACACGGTGCGACAGGCTGAAAG | 61.9 |  |
| Poh0114530.1 | *JAZ-2* | JAZ02F | AACGGCGAGGAAGGCAACGA | 60.5 | 185 |
|  |  | JAZ02R | AGGAACGGGTAGTCGGTGTAGC | 59.8 |  |
| Poh0024230.1 | *OPR-1* | OPR01F | TGCTCCTGACCGTGACTCGTA | 58.8 | 137 |
|  |  | OPR01R | GGTTGTGCTCTGGTTGTGATGC | 58.7 |  |
| Poh0281560.1 | *OPR-2* | OPR02F | CACAGGTCACAGAGGAGAGT | 54.0 | 192 |
|  |  | OPR02R | TACCAGCCAGAACCATAATAGC | 53.7 |  |
| Poh0127470.1 | *AP2/ERF-1* | AP2/ERF01F | AGAGACCAGCATCGGACATTAGAG | 58.3 | 113 |
|  |  | AP2/ERF01R | TCTGTACTTCGGACTTATCGGACT | 57.1 |  |
|  |  | AP2/ERF02R | GGCACCAGAGGCTCATTCAAGAC | 59.9 |  |
| Poh0232990.1 | *AP2/ERF-2* | AP2/ERF02F | TTGCGGTGAAGAAGGAGGAGAGTC | 60.6 | 220 |
|  |  | AP2/ERF02R | GCGGGCGAGTCAGGGAAGTTTA | 60.9 |  |
| Poh0291140.1 | *PAL-1* | PAL01F | GCAGACGGCAGTGAAGCAAGTGGT | 64.3 | 108 |
|  |  | PAL01R | AGCAGCAGGTCCTTCTCGCAG | 60.6 |  |
| Poh0244630.1 | *PAL-2* | PAL02F | GAGGCGGTGGAGATTCTGAAGC | 59.4 | 151 |
|  |  | PAL02R | CCTTGCCCATGAACAGCGTCTT | 59.7 |  |
| Poh0273640.1 | *2-OGD-1* | 2-OGD01F | CGGAGGCGATTGAAGGAGGAGT | 60.1 | 147 |
|  |  | 2-OGD01R | TGGAATGCGGCGTGTAGGTGAT | 60.6 |  |
| Poh0157150.1 | *2-OGD-2* | 2-OGD02F | GGAGGAGTGGCTATGGCGAACAAT | 61.3 | 133 |
|  |  | 2-OGD02R | AGGAATGCGGCGTGAAGGTGAT | 60.9 |  |
| Poh0291130.1 | *CHS-1* | CHS01F | GCTCGTCGGTGTTGTTCGTGTT | 60.3 | 109 |
|  |  | CHS01R | GGTCCGAAGCCGATGAAGAAGC | 59.9 |  |
| Poh0291180.1 | *CHS-2* | CHS02F | GCCGCAGTGGAACGAGATG | 56.9 | 160 |
|  |  | CHS02R | CACGAACAACACCGACGAGC | 58.0 |  |
| Poh0161990.1 | *UFGT-1* | UFDP01F | TCGTCGGTGCTGTATGTCTCCT | 59.2 | 182 |
|  |  | UFDP01R | CCTCTTCCCTTCGTCCGTTCCT | 59.7 |  |
| Poh0253860.1 | *UFGT-2* | UFDP02F | GCAGAGCCAGAACGCAGAAGATG | 60.5 | 173 |
|  |  | UFDP02R | AAGAGCCTCCGTTGCCGACT | 59.5 |  |
| Poh0204970.1 | *PnGAPDH* | PnGAPDH-1q5 | AGGAAGGACTCGCCTCTGGAAG | 59.5 | 142 |
|  |  | PnGAPDH-1q3 | CGATACTGATGCCGTCGTTGCC | 60.3 |  |
| Poh0314480.1 | *Actin-1* | PnAct1-qPCR1 | CGGAAACATCGTGCTGAGTGGAG | 60.2 | 185 |
|  |  | PnAct1-qPCR2 | ACATCTGCTGGAAGGTGCTGAGA | 60.0 |  |
| Poh0012540.1 | *β-tubulin-1* | PnTub1-qPCR1 | TCGTCGTACTTCGTGGAGTGGA | 59.2 | 160 |
|  |  | PnTub1-qPCR2 | TCCTGAACATCGCCGTGAACTG | 59.2 |  |
