# Supplementary material for: Metabolic profiling and gene expression analyses provide insights into cold adaptation of an Antarctic moss Pohlia nutans
Source: Front Plant Sci. 2022 Sep 13;13:1006991. doi: 10.3389/fpls.2022.1006991 (PMC9514047; doi:10.3389/fpls.2022.1006991)
Supplement: Supplementary file 8 [file Table_6.DOCX]

**Supplementary Table 6** Summary of data quality in transcriptome sequencing.

| **Sample name** | **Raw reads** | **Clean reads** | **UMI reads** | **Raw bases** | **Clean bases** | **Q20 (%)** | **Q30 (%)** | **UMI Clean reads (%)** | **Deduplicate Mapped UMI (%)** | **Deduplicated ratio (%)** |
| --- | --- | --- | --- | --- | --- | --- | --- | --- | --- | --- |
| Cold_0h_1 | 47,027,694 | 46,630,864 | 44,947,714 | 7.05G | 6.99G | 97.75 | 93.44 | 96.39 | 73.66 | 26.34 |
| Cold_0h_2 | 43,648,666 | 43,084,154 | 41,332,718 | 6.55G | 6.46G | 97.42 | 92.74 | 95.93 | 74.86 | 25.14 |
| Cold_0h_3 | 45,037,576 | 44,460,884 | 42,676,622 | 6.76G | 6.67G | 97.42 | 92.82 | 95.99 | 73.57 | 26.43 |
| Cold_6h_1 | 44,154,362 | 43,563,262 | 41,879,642 | 6.62G | 6.53G | 97.45 | 92.85 | 96.14 | 75.02 | 24.98 |
| Cold_6h_2 | 44,416,666 | 43,854,738 | 42,103,582 | 6.66G | 6.58G | 97.36 | 92.69 | 96.01 | 73.67 | 26.33 |
| Cold_6h_3 | 42,135,180 | 41,607,608 | 39,926,374 | 6.32G | 6.24G | 97.39 | 92.73 | 95.96 | 76.72 | 23.28 |
| Cold_24h_1 | 44,359,792 | 43,834,138 | 42,100,174 | 6.65G | 6.58G | 97.48 | 92.96 | 96.04 | 73.14 | 26.86 |
| Cold_24h_2 | 40,997,114 | 40,480,902 | 38,869,598 | 6.15G | 6.07G | 96.97 | 91.76 | 96.02 | 73.08 | 26.92 |
| Cold_24h_3 | 44,244,924 | 43,726,542 | 42,056,416 | 6.64G | 6.56G | 97.57 | 93.02 | 96.18 | 73.89 | 26.11 |
| Cold_60h_1 | 40,735,638 | 40,291,914 | 38,651,380 | 6.11G | 6.04G | 97.43 | 92.69 | 95.93 | 74.01 | 25.99 |
| Cold_60h_2 | 45,690,354 | 45,120,240 | 43,309,976 | 6.85G | 6.77G | 97.39 | 92.58 | 95.99 | 74.74 | 25.26 |
| Cold_60h_3 | 45,050,830 | 44,560,106 | 42,899,910 | 6.76G | 6.68G | 97.54 | 93.02 | 96.27 | 75.25 | 24.75 |

(1) Raw reads：The count of original sequence data.

(2) Clean reads：The count of the filtered sequencing data.

(3) UMI reads：The number of reads that match the specific unique molecular identifiers pattern.

(4) Raw/Clean bases：The data size of Raw/Clean reads.

(5) Q20/Q30：The percentages of the bases with phred values larger than 20 and 30 in the total reads obtained by sequencing, respectively.

(6) UMI Clean reads：Percentage of UMI reads in clean reads.

(7) Deduplicate Mapped UMI reads：The percentage of UMI reads after de duplication compared to the number of UMI reads on the reference genome.

(8) Deduplicated ratio：According to the comparison results and the weight removal percentage of unique molecular identifiers.
