# Supplementary material for: Metabolic profiling and gene expression analyses provide insights into cold adaptation of an Antarctic moss Pohlia nutans
Source: Front Plant Sci. 2022 Sep 13;13:1006991. doi: 10.3389/fpls.2022.1006991 (PMC9514047; doi:10.3389/fpls.2022.1006991)
Supplement: Supplementary file 9 [file Table_7.DOCX]

**Supplementary Table 7** The ratio of sequencing reads mapping to genome in different samples

| Sample name | Cold_0h_1 | Cold_0h_2 | Cold_0h_3 | Cold_6h_1 | Cold_6h_2 | Cold_6h_3 | Cold_24h_1 | Cold_24h_2 | Cold_24h_3 | Cold_60h_1 | Cold_60h_2 | Cold_60h_3 |
| --- | --- | --- | --- | --- | --- | --- | --- | --- | --- | --- | --- | --- |
| UMI reads | 44947714 | 41332718 | 42676622 | 41879642 | 42103582 | 39926374 | 42100174 | 38869598 | 42056416 | 38651380 | 43309976 | 42899910 |
| UMI mapped | 43396977 (96.55%) | 39912570 (96.56%) | 41221483 (96.59%) | 40590772 (96.92%) | 40637285 (96.52%) | 38676658 (96.87%) | 40335529 (95.81%) | 37581342 (96.69%) | 40764722 (96.93%) | 37546192 (97.14%) | 42093697 (97.19%) | 41691089 (97.18%) |
| Multiple mapped | 3922997 (8.73%) | 3726430 (9.02%) | 3812773 (8.93%) | 3348809 (8%) | 3459526 (8.22%) | 3111241 (7.79%) | 3722179 (8.84%) | 3099105 (7.97%) | 3572554 (8.49%) | 3158379 (8.17%) | 3594333 (8.3%) | 3473054 (8.1%) |
| Uniquely mapped | 39473980 (87.82%) | 36186140 (87.55%) | 37408710 (87.66%) | 37241963 (88.93%) | 37177759 (88.3%) | 35565417 (89.08%) | 36613350 (86.97%) | 34482237 (88.71%) | 37192168 (88.43%) | 34387813 (88.97%) | 38499364 (88.89%) | 38218035 (89.09%) |
| Read-1 | 19733966 (43.9%) | 18114196 (43.83%) | 18731098 (43.89%) | 18635375 (44.5%) | 18617983 (44.22%) | 17804896 (44.59%) | 18319670 (43.51%) | 17295293 (44.5%) | 18607290 (44.24%) | 17209063 (44.52%) | 19270083 (44.49%) | 19119461 (44.57%) |
| Read-2 | 19740014 (43.92%) | 18071944 (43.72%) | 18677612 (43.77%) | 18606588 (44.43%) | 18559776 (44.08%) | 17760521 (44.48%) | 18293680 (43.45%) | 17186944 (44.22%) | 18584878 (44.19%) | 17178750 (44.45%) | 19229281 (44.4%) | 19098574 (44.52%) |
| Reads map to '+' | 19724402 (43.88%) | 18082966 (43.75%) | 18693903 (43.8%) | 18612249 (44.44%) | 18580879 (44.13%) | 17774690 (44.52%) | 18301131 (43.47%) | 17232941 (44.34%) | 18587005 (44.2%) | 17181778 (44.45%) | 19236044 (44.41%) | 19096332 (44.51%) |
| Reads map to '-' | 19749578 (43.94%) | 18103174 (43.8%) | 18714807 (43.85%) | 18629714 (44.48%) | 18596880 (44.17%) | 17790727 (44.56%) | 18312219 (43.5%) | 17249296 (44.38%) | 18605163 (44.24%) | 17206035 (44.52%) | 19263320 (44.48%) | 19121703 (44.57%) |
| Non-splice reads | 25721125 (57.22%) | 23625503 (57.16%) | 24289144 (56.91%) | 25486537 (60.86%) | 25432614 (60.4%) | 24177149 (60.55%) | 24840845 (59%) | 23479350 (60.41%) | 25472992 (60.57%) | 22726158 (58.8%) | 25551021 (59%) | 25198712 (58.74%) |
| Splice reads | 13752855 (30.6%) | 12560637 (30.39%) | 13119566 (30.74%) | 11755426 (28.07%) | 11745145 (27.9%) | 11388268 (28.52%) | 11772505 (27.96%) | 11002887 (28.31%) | 11719176 (27.87%) | 11661655 (30.17%) | 12948343 (29.9%) | 13019323 (30.35%) |

The statistical results of the comparison are as follows:

(1) UMI reads, the statistics of Clean UMI sequencing reads after quality trimming (Clean UMI data).

(2) UMI mapped, the statistics of UMI reads that can be mapped to reference genome.

(3) Multiple mapped, the statistics of UMI reads with multiple alignment positions on the reference sequence.

(4) Uniquely mapped, the statistics of UMI reads with unique alignment position on the reference sequence.

(5) Reads map to '+'，Reads map to '-', the statistics of sequence alignment to positive and negative chains on the genome.
